# Intraoperative Ultrasound‐Guided Repair of Penile Fracture: A Case Report

**DOI:** 10.1155/criu/3418235

**Published:** 2026-09-08

**Authors:** Hoi-Lung Wong, Omar Wai-Kiu Tsui, Brian Sze-Ho Ho, Ada Tsui-Lin Ng

**Affiliations:** ^1^ Division of Urology, Department of Surgery, Queen Mary Hospital, Hong Kong, China, qmh.org.hk; ^2^ Division of Urology, Department of Surgery, The University of Hong Kong, Hong Kong, China, hku.hk

**Keywords:** intraoperative USG (IO-USG), minimally invasive surgery, penile fracture, repair of penile fracture, tunica albuginea

## Abstract

Penile fracture is a rare urological emergency resulting from rupture of the tunica albuginea during blunt trauma to an erect penis, typically during sexual intercourse. While immediate surgical repair is associated with superior outcomes compared with conservative management, delayed treatment can lead to significant long‐term complications including penile curvature in 30% of cases and erectile dysfunction in 50%–62% of cases. We present the case of a 22‐year‐old patient who sustained a penile fracture while manipulating his erect penis. Clinical examination revealed penile swelling, bruising, and a 60° leftward curvature. Intraoperative ultrasound (IO‐USG) with a linear probe precisely localized a tunica albuginea defect in the right corpus cavernosum, enabling a minimally invasive 1.5‐cm transverse incision directly over the fracture site. This approach avoided complete penile degloving, reduced operative time and blood loss, and facilitated complete clot evacuation and defect closure using interrupted 3‐0 PDS sutures. Postoperative recovery was uneventful, with the patient discharged the following day and reporting normal erectile function and no penile deformity at the 3‐month follow‐up. A literature review confirms that while preoperative imaging is commonly utilized for surgical planning, IO‐USG guidance for real‐time fracture localization and incision placement remains rarely reported. Although MRI and CT offer superior soft tissue resolution, they are limited by availability, time constraints, and radiation exposure. IO‐USG is readily accessible and noninvasive; although operator‐dependent, it provides high‐resolution real‐time guidance that enables smaller incisions, minimizes postoperative complications such as glans numbness, and optimizes functional outcomes. We conclude that IO‐USG–guided repair of penile fracture facilitates rapid identification of the injury site, accurate assessment of hematoma burden, and targeted surgical approach that avoids penile degloving while ensuring complete repair.

Keynote Message

Penile fracture is an uncommon urological emergency that requires urgent medical attention and surgical treatment. We present a case of surgical exploration of a penile fracture with the aid of intraoperative ultrasound (IO‐USG), which offers a viable alternative to traditional complete penile degloving, directly optimizing functional and cosmetic outcomes.

## 1. Introduction

Penile fracture is the disruption of the tunica albuginea with rupture of the corpus cavernosum and an uncommon urological emergency that requires urgent medical attention and surgical treatment. The literature has shown that immediate surgical repair yields better outcomes than conservative management, with late‐onset complications such as penile curvature occurring in 30% of cases and erectile dysfunction in 50%–62% of cases [1]. Most of these injuries result from direct blunt trauma to the penis during sexual intercourse. Severe blunt trauma to an erect penis can cause rupture of the tunica albuginea, a condition termed penile fracture.

## 2. Case Presentation

A 22‐year‐old patient with good past health presented to emergency department for painful penile swelling. He had penile erection in the morning. He attempted to manipulate his penis from sideways to central position and subsequently he heard a pop sound. He experienced detumescence of erection followed by severe penile pain and swelling. There was no gross hematuria, and he was able to void.

**Figure 1:**
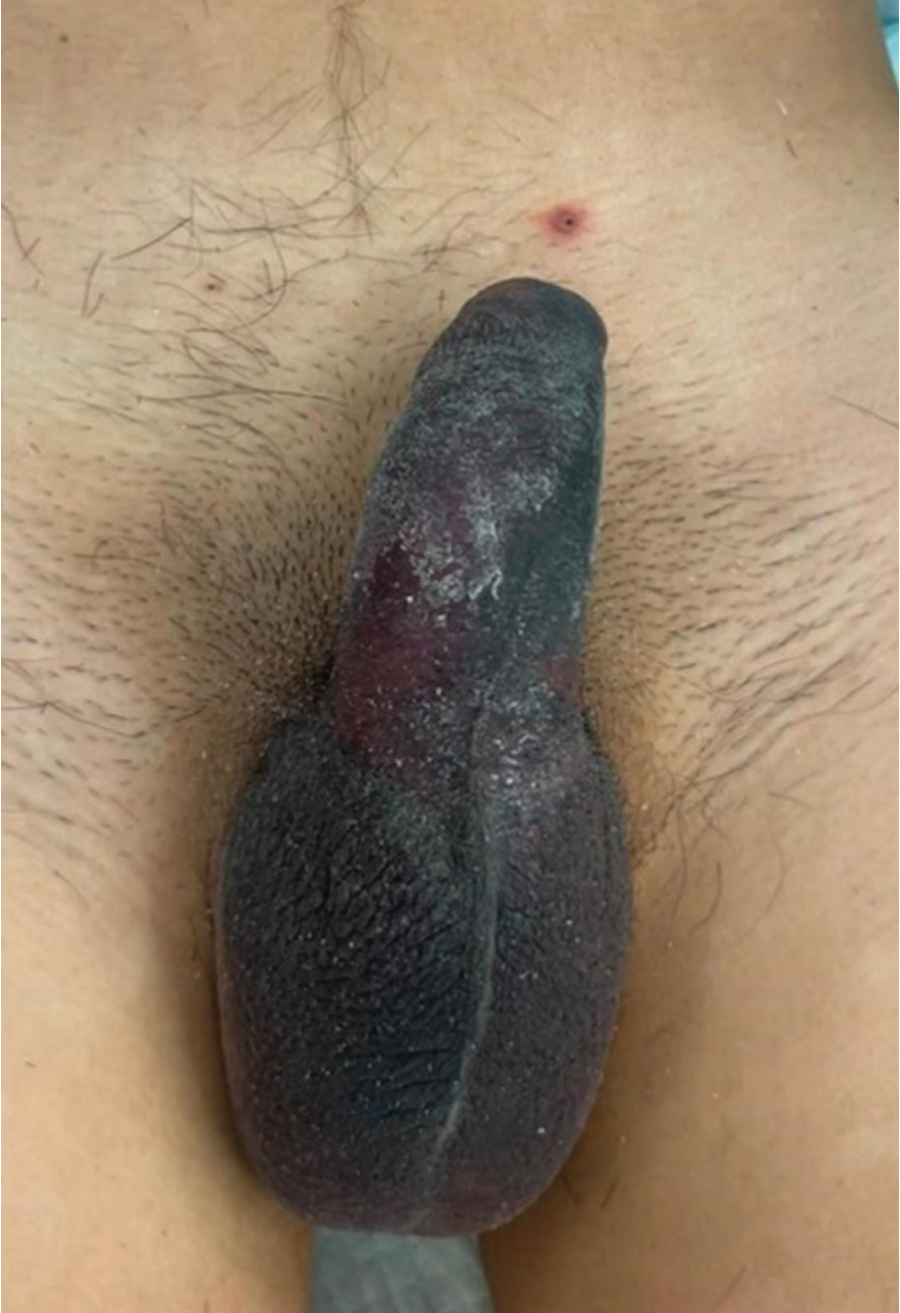
The penis was flaccid, with marked swelling and bruising observed.

On physical examination, the penis was flaccid, with marked swelling and bruising observed. There was a penile deformity with a curvature of approximately 60° to the left side. On palpation, a hematoma was palpable at the proximal base of the ventral side of the penis. The foreskin was retractable, the glans appeared healthy, and no blood was observed from the urethral meatus. The bilateral testes were normal, with no scrotal swelling or hematoma noted (Figure 1). Emergency bedside preoperative ultrasound was performed before sending to operation theater. The patient was offered surgical exploration and repair, along with flexible cystoscopy, under general anesthesia.

**Figure 2:**
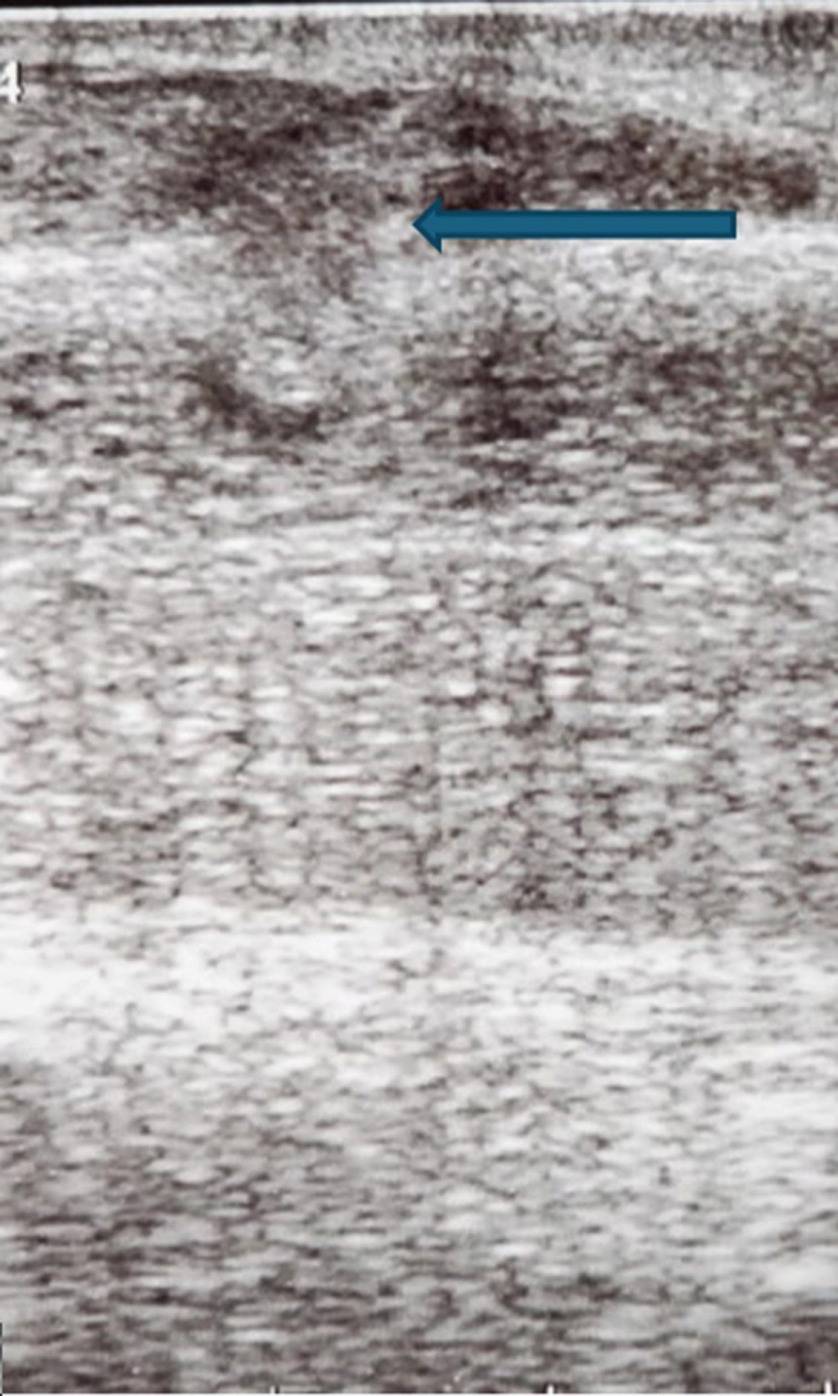
The ultrasound demonstrated a defect in the tunica albuginea of the right corpus cavernosum (the blue arrow).

**Figure 3:**
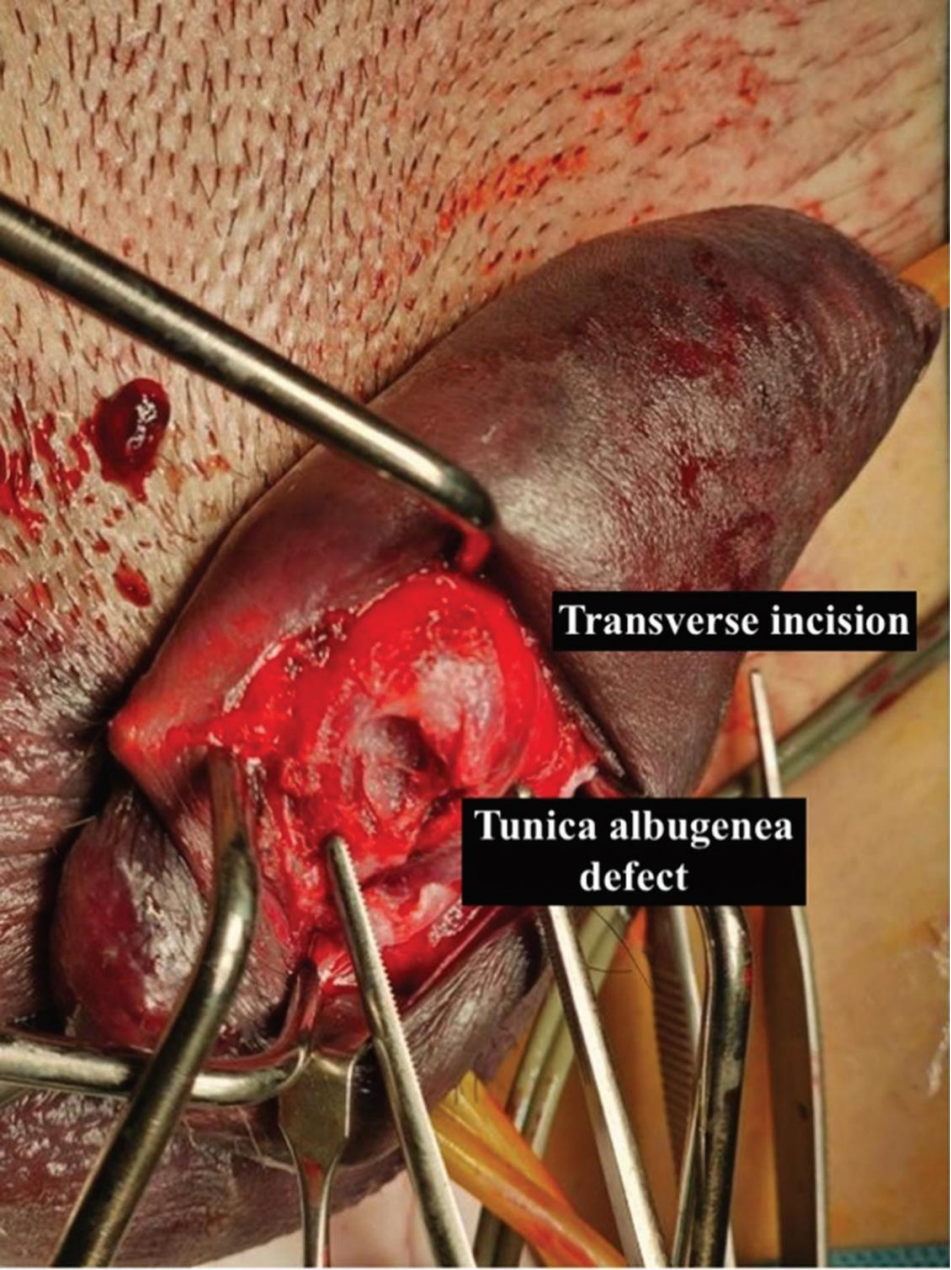
A small incision was made at the location identified by the ultrasound. The defect in the tunica was closed with suture.

The patient was then transferred to the operating theater for surgical exploration. Flexible cystoscopy revealed a normal urethra without signs of injury. On palpation, a 5‐cm hematoma was identified over the right proximal penile shaft. IO‐USG with a linear probe demonstrated a defect in the tunica albuginea of the right corpus cavernosum (Figure 2). A transverse incision of 1.5 cm was made over the right proximal penile shaft at the location identified by the USG. The hematoma was opened, and clots were evacuated (Figure 3) The wound was irrigated with normal saline, and the defect was identified and cleaned. The defect in the tunica albuginea was closed using 3‐0 PDS sutures in an interrupted fashion. Artificial erection was induced using a butterfly needle and normal saline, which confirmed no leakage and the absence of penile curvature or deformity. The dartos layer was closed with 3‐0 Vicryl sutures, and the skin was closed with Vicryl Rapide.

Postoperatively, the penile swelling and pain improved, and the patient was discharged the following day. At the 3‐month follow‐up, the patient reported normal erectile function and ejaculation. There were no complaints of penile pain, numbness, curvature, or deformity.

## 3. Discussion

### 3.1. Literature Review

Based on multiple case reports (Table 1), most cases utilized preoperative imaging—such as USG, computed tomography (CT), or magnetic resonance imaging (MRI)—for surgical planning, yet only one case reported the use of in IO‐USG for real‐time guidance during incision and clot evacuation. Traditional penile fracture repair often requires degloving of the penis, which increases both blood loss and operative time. In contrast, using IO‐USG allowed us to precisely localize the fracture site and assess the hematoma size, enabling a smaller, more targeted incision. IO‐USG enables a more accurate, less invasive, and safer repair, as it can guide incision size/location, repair technique, and completeness of closure. In our case, IO‐USG not only guided the chief surgeon to the exact fracture location but also provided real‐time evaluation of the blood clot burden. This informed the optimal incision site, minimized incision size, and facilitated complete fracture repair and clot evacuation.

**Table 1:** Literature review table.

PMID	Age (years)	Preoperative imaging	On‐table image guidance	Surgical approach	Clinical outcomes	Complications	Incision length (cm)/degloving
27072182 [2]	21	MRI	Nil	Minimally invasive ventral approach	Not mentioned	Not mentioned	Around estimated 4 cm (from clinical picture in the case report)
28942396 [3]	47	USG	Yes, USG	Limited vertical incision distal to penoscrotal junction	Complete healing of the urethra	N/A	N/A
30556992 [4]	28	CT	Nil	Degloving	Good erection with no angular deformity or plaque formation after 3 months follow‐up	N/A	Degloving
31543948 [5]	32	N/A	Nil	Degloving	Proper erectile function, no deformity, no pain during sexual intercourse, no voiding difficulties	N/A	Degloving
33426494 [6]	25	MRI	Nil	Transperineal	No perineal pain, dysuria or erectile dysfunction	N/A	N/A
33532436 [7]	25	USG	Nil	Degloving	Voiding well with no erectile dysfunction, no pain or chordee on erection	Require psychiatric consultation	Degloving
33645282 [8]	36	N/A	Nil	Subcoronal circumferential incision	IIEF5 score 22; normal voiding and sexual function after 1 year follow‐up	N/A	Degloving
35983255 [9]	34	N/A	Nil	Degloving	Normal voiding and erectile function for 2 years follow‐up	N/A	Degloving
36204410 [10]	46	Urethrography with retrograde urethrogram fluoroscopy	Nil	Subcoronal circumferential incision	No gross hematuria, swelling or edema at the penile soft tissue	Urethral stricture at the cavernous part with no contrast leakage	Degloving
36275834 [11]	29	USG	Nil	Scrotal incision	Normal erection after 8 weeks	N/A	N/A
36353400 [12]	45	N/A	Nil	Penoscrotal approach	Voiding well, no erectile dysfunction, no penile curvature, or nodule	N/A	N/A
36523731 [13]	30	N/A	Nil	Degloving	No recurrence of penile fracture	Penile edema	Degloving
36605679 [14]	61	MRI	Nil	Transverse incision	No discoloration of swelling of the penile shaft, return of erectile function; no urethral stenosis or injury	Residual edema	5 cm
36915707 [15]	37	N/A	Nil	Subcoronal circumferential incision	Good sexual outcome, no difficulty in voiding	Bluish color seen at distal penis	Degloving
37126924 [16]	36	Retrograde urethrography, USG, MRI	Nil	Degloving	Resumed sexual life, no induration	N/A	Degloving
37399592 [17]	27	USG	Nil	Subcoronal circumferential incision	Normal voiding and sexual function	N/A	Degloving
37886346 [18]	45	Retrograde urethrography, USG	Nil	Degloving	Unchanged urinary flow; normal and painless erections	N/A	Degloving
38426598 [19]	30–40	MRI	Nil	Perineal surgical approach	Normal preinjury erectile function	N/A	N/A
38615465 [20]	27	USG	Nil	Degloving	Full erectile function	N/A	Degloving
40656391 [21]	38	N/A	Nil	Degloving	No penile deformity, erectile dysfunction, or dyspareunia	N/A	Degloving
40786337 [22]	55	USG, MRI	Nil	Degloving	IIEF5 score 24; normal erectile function	N/A	Degloving
40989260 [23]	27	MRI	Nil	Perineal surgical approach	Not mentioned	Not mentioned	N/A
42086364 [24]	41	USG	Nil	Penoscrotal incision	Sexual function and micturition restored at post op 3 months	Residual leakage at anterior urethera; phimosis due to prolonged foreskin edema	5 cm

### 3.2. Main Discussion

Fracture of the penis is a relatively rare condition, defined as the rupture of the tunica albuginea due to trauma to an erect penis. The tissue integrity of the tunica albuginea is compromised by a sudden increase in intracorporeal pressure. The injury is typically caused by direct blunt force or sudden bending of the penis.

Common clinical manifestations of penile fracture include sudden onset of pain, penile swelling, and ecchymosis following blunt trauma to the erect penis. It may also be associated with detumescence, voiding difficulties, penile swelling, and deviation. The swelling and hematoma can spread to the scrotum, perineum, and lower abdominal wall within the Colles fascia.

The diagnosis is usually based on characteristic clinical presentation and physical examination. Imaging studies may be considered if the presentation and physical findings are atypical. USG, color‐Doppler USG, cavernosography, angiography, and MRI can be utilized for further assessment.

USG examination is noninvasive and allows for quick assessment of the injured site. In our case, we used a linear probe USG to guide the site of incision. During the operation, the USG revealed a defect in the tunica albuginea of the right corpus cavernosum. A 1.5‐cm transverse incision was made over the right proximal penile shaft, precisely at the location identified by the USG. This approach allowed for a smaller incision, avoided the full penile degloving procedure and real‐time evaluation of effectiveness of blood clot removed, which is associated with risks such as penile numbness and a longer recovery time before resumption of sexual activity [25]. However, ultrasound is operator‐dependent, and patients may experience pain during manipulation in the acute setting.

Unlike preoperative bedside ultrasonography, which is often hindered by patient discomfort, involuntary guarding, and suboptimal penile positioning, IO‐USG is performed under general anesthesia, offering distinct diagnostic advantages. The anesthetized state effectively eliminates patient movement and pelvic muscle tension, providing a static and stable acoustic window that minimizes motion artifacts and enhances image resolution. Furthermore, the complete absence of pain abolishes pain‐induced penile reflexes or spasms, allowing the operator to manipulate the penis gently and position it in optimal multiplanar orientations—including longitudinal, transverse, and oblique views—without causing patient distress or compromising the examination. This controlled environment facilitates a more thorough and systematic assessment of the tunical defect, enabling precise measurement of the tear′s dimensions and accurate delineation of its spatial relationship to the urethra and neurovascular bundles. Consequently, IO‐USG not only surpasses the diagnostic accuracy of the bedside study but also serves as an indispensable intraoperative navigational tool, ensuring that the surgical repair is tailored precisely to the individual anatomy with minimal invasiveness. Furthermore, IO‐USG allows immediate postrepair verification of closure completeness and exclusion of residual bleeding.

Diagnostic cavernosography or MRI can also be used as diagnostic tools for penile fracture. However, these methods are limited by availability and the time‐consuming nature of the procedures. Surgical repair should be performed promptly, as delays can lead to poorer healing, increased fibrosis, and a higher risk of erectile dysfunction. Several studies have demonstrated significant differences in functional outcomes between immediate and delayed repairs of penile fractures [26–28].

In our case, USG facilitated a prompt diagnosis, localized the site of the defect, and allowed for a smaller wound incision, thereby maximizing the chance of a good long‐term clinical outcome. Penile fracture is a condition that requires accurate diagnosis for effective treatment planning. Although MRI is a radiation‐free alternative and the best radiological tool in terms of soft tissue resolution compared with ultrasound, it is not always readily available, and the scanning process is time‐consuming [2]. CT‐guided minimally invasive penile fracture repair was also reported in the literature. It also allows high‐resolution images and allows detecting the tear location precisely. However, it is still limited by the availability and image‐processing time. In contrast, USG is readily accessible, provides high‐resolution images, and offers key information during surgical exploration. Table 2 shows the comparison of different imaging‐guided repair of penile fracture.

**Table 2:** Comparison of different image‐guided repair of penile fracture.

Author	Wong et al.	Yan et al.	Giovanni Rosi et al.
Modality used	USG	CT	MRI
Localization of injury site	Yes	Yes	Yes
Radiation	No	Yes	No
Imaging resolution	Good	Excellent	Excellent
Operator dependent	Yes	No	No
Cost	Low	Moderate	High
Scanning time	Fast	Moderate	Slow
Able to use in operation theater	Yes	Limited by availability of hospital	No

Regarding the incision for surgical exploration, there are three main possible approaches: penile degloving via an incision under the balanopreputial groove, the penoscrotal approach, or an elective approach involving an incision directly over the fracture site. Reichenbach et al. [25] reported a significantly higher rate of postsurgery glans numbness following penile degloving compared with the elective approach. For the elective approach, palpating the fracture line and determining the location of the incision may be challenging and often requires experienced operators. However, with the aid of IO‐USG guidance, the fracture site can be easily localized, allowing surgeons to avoid the use of the degloving incision while achieving accurate results.

## 4. Conclusion

IO‐USG–guided repair of fracture penis allowed a quick identification of the injured site, real‐time assessment of fracture and hematoma, and a smaller incision for surgical repair, and obviated the need to deglove the entire penis. However, given the short follow‐up interval in our patient, additional longitudinal assessment is warranted to comprehensively evaluate the long‐term prognosis of penile fracture.

## Funding

No funding was received for this manuscript.

## Disclosure

All authors have read and approved the final version of the manuscript. Hoi‐Lung Wong had full access to all of the data in this study and takes complete responsibility for the integrity of the data and the accuracy of the data analysis. We have not used any artificial intelligence.

## Consent

Informed consent has been obtained from the patient.

## Conflicts of Interest

The authors declare no conflicts of interest.

## Supporting information


**Supporting Information** Additional supporting information can be found online in the Supporting Information section. The supporting file includes a file of Supporting Information, which includes details of data availability, disclosures, acknowledgements, and declaration.

## Data Availability

The data that support the findings of this study are available on request from the corresponding author. The data are not publicly available due to privacy or ethical restrictions.
