# Supplementary material for: Intraoperative Ultrasound‐Guided Repair of Penile Fracture: A Case Report
Source: Case Rep Urol. 2026 Sep 8;2026:3418235. doi: 10.1155/criu/3418235 (PMC13551490; doi:10.1155/criu/3418235)
Supplement: Supplementary file 1 [file CRIU-2026-3418235-s001.docx]

**Data availability:**

The data that support the findings of this study are available on request from the corresponding author. The data are not publicly available due to privacy or ethical restrictions.

**Disclosures:**

(i) Conflict of interest: The authors declare no conflict of interest.
(ii) Approval of the research protocol by an institutional reviewer board: Not applicable
(iii) There are no fundings in this case report.
(iv) Informed consent: Informed consent has been obtained from patient
(v) Registry and the registration no. of the study/trial: Not applicable
(vi) We have not used any artificial intelligence

**Acknowledgements:** Nil

**Declaration:**

All authors have read and approved the final version of the manuscript. Hoi-lung Wong had full access to all of the data in this study and takes complete responsibility for the integrity of the data and the accuracy of the data analysis.

**References:**

1. Amer T, Wilson R, Chlosta P, AlBuheissi S, Qazi H, Fraser M, Aboumarzouk OM. Penile Fracture: A Meta-Analysis. Urol Int. 2016;96(3):315-29. doi: 10.1159/000444884. Epub 2016 Mar 9. PMID: 26953932.
2. Reichenbach A, Gayrel P, Nicolas Morel Journel, Carnicelli D, Vedrine N. et al. Surgical management for penile fracture: A retrospective, observational, multicenter study. The French Journal of Urology. 2024 Apr 1;34(4):102590–0.
3. Hinev A. Fracture of the penis: treatment and complications. Acta medica Okayama. 2000 Oct;54(5):211–6.
4. Gedik A, Kayan D, Yamiş S, Yılmaz Y, Bircan K. The diagnosis and treatment of penile fracture: our 19-year experience. Ulusal travma ve acil cerrahi dergisi = Turkish journal of trauma & emergency surgery: TJTES [Internet]. 2011 Jan 1;17(1):57–60.
5. El Atat R, Sfaxi M, Benslama MR, Amine D, Ayed M, et al. Fracture of the Penis: Management and Long-Term Results of Surgical Treatment. Experience in 300 Cases. Journal of Trauma and Acute Care Surgery. 2008 Jan ;64(1):121. ‌
6. Rosi G, Fontanella P, Venzi G, Jermini F, Del Grande F. 3T MR-guided minimally-invasive penile fracture repair. Archivio italiano di urologia, andrologia : organo ufficiale [di] Societa italiana di ecografia urologica e nefrologica. 2016;88(1):68–9.
7. Czarnecki O, von Stempel CB, Sangster P, Walkden M. Microbubble-enhanced ultrasound to demonstrate urethral transection in a case of penile fracture. BMJ Case Rep. 2017 Sep 23;2017:bcr2017220073. doi: 10.1136/bcr-2017-220073. PMID: 28942396; PMCID: PMC5747639.
8. Yan C, Liang BX, Huang HB, Liang BR, Zhou Z, Wang LJ, Yang ZQ, Xian SX. CT-guided minimally-invasive penile fracture repair. Int Braz J Urol. 2019 Jan-Feb;45(1):183-186. doi: 10.1590/S1677-5538.IBJU.2018.0525. PMID: 30556992; PMCID: PMC6442154.
9. Mercado-Olivares F, Grandez-Urbina JA, Farfan-Daza G, Pacheco-Sauñe J, Nuñez-Bragayrac L. Case Report: Double penile fracture. F1000Res. 2018 Nov 21;7:1828. doi: 10.12688/f1000research.16452.2. PMID: 31543948; PMCID: PMC6733384.
10. Harada J, Shida Y, Gono S, Masato M, Yuno T, Hakariya T, Kihara T, Maeda K, Miyata Y, Sakai H. Rare case of penile fracture caused by an injury to the crus penis: Delayed repair using the transperineal approach. IJU Case Rep. 2020 Nov 17;4(1):36-38. doi: 10.1002/iju5.12232. PMID: 33426494; PMCID: PMC7784756.
11. Bhardwaj R, Gaurav G, Alam S. Penile Fracture during masturbation - a case report. J Family Med Prim Care. 2020 Nov 30;9(11):5790-5792. doi: 10.4103/jfmpc.jfmpc_1160_20. PMID: 33532436; PMCID: PMC7842420.
12. Mathew J, Parmar K, Chandna A, Kumar S. Penile fracture associated with complete urethra and bilateral corpora cavernosa transection. Ann R Coll Surg Engl. 2021 Mar;103(3):e88-e90. doi: 10.1308/rcsann.2020.7042. PMID: 33645282; PMCID: PMC9158049.
13. Makama BS, Haruna L, Stephen Y, Aminu U. Penile Fracture with Complete Urethral Rupture and Urine Extravasation: A Case Report. J West Afr Coll Surg. 2021 Apr-Jun;11(2):31-34. doi: 10.4103/jwas.jwas_18_22. Epub 2022 Jul 12. PMID: 35983255; PMCID: PMC9380785.
14. Sulistio PA, Prijambodo. Urethrography for diagnostic of penile fracture with ruptured anterior urethra and right corpus cavernosum. Radiol Case Rep. 2022 Sep 30;17(12):4694-4699. doi: 10.1016/j.radcr.2022.08.104. PMID: 36204410; PMCID: PMC9529555.
15. Kohpe Kapseu S, Traore TM. Penile fracture revealed by acute painless swollen scrotum: Case report. Urol Case Rep. 2022 Oct 12;45:102259. doi: 10.1016/j.eucr.2022.102259. PMID: 36275834; PMCID: PMC9579785.
16. Joshi BM, Ranjan SK, Jain M, Kumar A. Penile Fracture with Urethral Rupture: The Feasibility of Repair through Penoscrotal Approach. J Emerg Trauma Shock. 2022 Jul-Sep;15(3):149-151. doi: 10.4103/jets.jets_154_21. Epub 2022 Sep 28. PMID: 36353400; PMCID: PMC9639730.
17. Krishnani H, Anjankar A, Kakar G, Sharma S, Kumar A. Penile Fracture With Urethral Bleeding: A Urological Emergency. Cureus. 2022 Nov 11;14(11):e31373. doi: 10.7759/cureus.31373. PMID: 36523731; PMCID: PMC9744420.
18. Yokoyama S, Tsuboi I, Ogawa K, Yoshioka S, Kobayasi Y, Nakajima H, Nagami T, Yamasaki S, Wada K. Isolated rupture of the corpus spongiosum with urethral injury diagnosed by pre-surgical MRI. IJU Case Rep. 2022 Nov 22;6(1):70-72. doi: 10.1002/iju5.12559. PMID: 36605679; PMCID: PMC9807332.
19. Nandana PI, Putra Nugraha TW. Penile fracture with urethral injury: A case report. Urol Case Rep. 2023 Feb 27;47:102365. doi: 10.1016/j.eucr.2023.102365. PMID: 36915707; PMCID: PMC10006510.
20. Ngowi BN, Bright F, Lazaro E, Lodhia J, Kimolo M, Mbwambo OJ. Triple penile fracture: A rare case report and review of literature. Int J Surg Case Rep. 2023 May;106:108245. doi: 10.1016/j.ijscr.2023.108245. Epub 2023 Apr 22. PMID: 37126924; PMCID: PMC10172890.
21. Kajerero V, Sukunala O, Rugakingira RA, Mkinga R, Mbwambo OJ, Tairo E. Penile fracture following sexual intercourse; A case report and literature review. Int J Surg Case Rep. 2023 Jul;108:108415. doi: 10.1016/j.ijscr.2023.108415. Epub 2023 Jun 20. PMID: 37399592; PMCID: PMC10382743.
22. Fernandes PF, Ferreira AM, Azinhais P, Figueiredo A. A penile fracture hidden behind a urethral bleeding. Urol Case Rep. 2023 Oct 16;51:102587. doi: 10.1016/j.eucr.2023.102587. PMID: 37886346; PMCID: PMC10598488.
23. Buckland BC, Heijkoop B, Tronidjaja J, Chong PG. Diagnostic precision in proximal penile fracture: the use of MRI and the perineal approach. ANZ J Surg. 2024 May;94(5):961-962. doi: 10.1111/ans.18915. Epub 2024 Mar 1. PMID: 38426598.
24. Gazzah W, Najjai Y, Lahouar R, Mzoughi M, Mansi Z, Braiek S. Delayed surgical repair in double penile fracture: Insights and outcomes: A case report. Int J Surg Case Rep. 2024 May;118:109623. doi: 10.1016/j.ijscr.2024.109623. Epub 2024 Apr 6. PMID: 38615465; PMCID: PMC11033179.
25. Haider SM, Khan MS, Amir M, Zaidi AR. Accidental Penile Trauma Resulting in a Fracture: A Case Report. Cureus. 2025 Jun 12;17(6):e85860. doi: 10.7759/cureus.85860. PMID: 40656391; PMCID: PMC12255469.
26. Safi A, Khera JS, Furtado N, Peters B. Penile Fracture in a 55-Year-Old Man: A Report of a Rare Case. Cureus. 2025 Jul 11;17(7):e87730. doi: 10.7759/cureus.87730. PMID: 40786337; PMCID: PMC12335856.
27. Welsh EM, Park J, Clark JY. Equivocal penile fracture clarified by MRI. Urol Case Rep. 2025 Sep 1;63:103196. doi: 10.1016/j.eucr.2025.103196. PMID: 40989260; PMCID: PMC12451281.
28. Samaras A, Aufderklamm S, Amend B, Stühler V, Stenzl A, Tsaur I, Rausch S. Penile fracture and concomitant severe urethral trauma-a case report and discussion of surgical approach. Can J Urol. 2026 Apr 15;33(2):477-481. doi: 10.32604/cju.2025.068588. PMID: 42086364.
